# Developing a primary care research agenda through collaborative efforts – a proposed “6E” model

**DOI:** 10.1186/s12930-014-0017-9

**Published:** 2014-12-20

**Authors:** Ngiap Chuan Tan, Chirk Jenn Ng, Mitchell Rosemary, Khan Wahid, Lee Gan Goh

**Affiliations:** SingHealth Polyclinics, Singapore, 167, Jalan Bukit Merah, #15-10, Connection One, Tower 5, Singapore, 538134 Singapore; Department of Primary Care Medicine, Faculty of Medicine, University of Malaya, 50603 Kuala Lumpur, Malaysia; Mitchells Clinic (Family Practice), 4th floor Tappoos City Mall, Suva, Fiji; JP Bayly Clinic, Suva, Fiji; Division of Family Medicine, National University Hospital System, NUHS Tower Block, 1E, Kent Ridge Road, Level 9, Singapore, 119 228 Singapore

**Keywords:** Primary care, Research agenda, Collaboration, Workshop, process, Model

## Abstract

**Background:**

Primary care research is at a crossroad in South Pacific. A steering committee comprising a member of WONCA Asia Pacific Regional (APR) council and the President of Fiji College of General Practitioners garnered sponsorship from Fiji Ministry of Health, WONCA APR and pharmaceutical agencies to organize the event in October 2013. This paper describes the processes needed to set up a national primary research agenda through the collaborative efforts of local stakeholders and external facilitators using a test case in South Pacific.

**Method:**

The setting was a 2-day primary care research workshop in Fiji. The steering committee invited a team of three external facilitators from the Asia-Pacific region to organize and operationalize the workshop. The eventual participants were 3 external facilitators, 6 local facilitators, and 29 local primary care physicians, academics, and local medical leaders from Fiji and South Pacific Islands. Pre-workshop and main workshop programs were drawn up by the external facilitators, using participants’ input of research topics relating to their local clinical issues of interest. Course notes were prepared and distributed before the workshop. In the workshop, proposed research topics were shortlisted by group discussion and consensus. Study designs were proposed, scrutinized, and adopted for further research development.

**Results:**

The facilitators reviewed the processes in setting the research agenda after the workshop and conceived the proposed 6E model. These processes can be grouped for easy reference, comprising the pre-workshop stages of “entreat”, “enlist”, “engage”, and the workshop stages of “educe”, “empower”, and “encapsulate”.

**Conclusion:**

The 6E model to establish a research agenda is conceptually logical. Its feasibility can be further tested in its application in other situation where research agenda setting is the critical step to improve the quality of primary care.

## Introduction

Effective and efficient primary care in a healthcare system is fundamental towards improving global health and reduces healthcare expenditure [1]. Naturally research in primary care allows the measurements of these attributes and provides a beacon to determine its direction towards achieving the desired goal of providing quality healthcare to the population. However research itself requires resources such as infrastructure, facilities, expertise and funding, which are often lacking in low and middle income countries. Strategic directives are thus necessary to optimize the limited resources in these countries for primary care research. This includes identifying the key healthcare issues in the local population, evaluating their clinical relevance and significance and collating them into a community-centric primary care research agenda. Such an agenda will serve as a platform to engage the local researchers and the health policy makers to review, deliberate and prioritize resources that is needed to support any research endeavors in the local primary care community. Drawing up a primary care research agenda requires careful planning and organization. Nonetheless, there is a dearth of information on the processes that are effective in its development.

Fiji and other South Pacific nations are developing countries. Strengthening the health system and primary care in the Western Pacific countries is one of the resolutions set by the World Health Organization (WHO) [2]. Primary and preventative healthcare services provide key healthcare services to the local populations on these island nations and research would have been instrumental to measure its effectiveness and efficiency [3]. However in 2004, Cuboni et al reported that health research in the Pacific was hithertho driven by non-Fijian researchers, with little local healthcare professionals’ involvement, processed the data and published them externally and brought meek benefits to the communities [4].

Five years later in 2009, the inaugural National Health Systems Research Workshop was organized in conjunction with the WHO, Ministry of Health Fiji and the Fiji School of Medicine [5]. This workshop was executed like a typical scientific meeting in which participants were invited to present their research papers for peer review on-site. A total of 27 papers covering oral health, nursing to human resource, including only a single paper on primary care were presented. A SWOT (Strength, Weakness, Opportunities and Threats) analysis of the workshop was performed which reported heightened participants’ interests in research, predominance of public health topics, lack of awareness of local research resources and recommended the need to engage the healthcare professionals in the private sector. There was no further progress report in the aftermath of this workshop. The absence of a focused research agenda and definitive action plan could explain the paucity of progress to bring this initiative forward.

A fresh initiative to accelerate the momentum of research in Fiji was mooted in 2012 when discussion began at the WONCA Asia Pacific Regional (APR) Conference in Jeju in South Korea to sponsor a research workshop to train primary healthcare professionals in Fiji and neighboring South Pacific nations. With the support and sponsorship of the Fiji Ministry of Health (MOH), the Fiji College of General Practitioners (FCGP) and WONCA APR, the two days “Primary Care Research Workshop” took place in October 2013.

### Aim

This article aims to present a proposed 6E model to describe the processes needed to develop the primary care research agenda, which is to address the health care needs of the local populations and build the research capacity of the local research communities, through increasing the quantity and quality of research output and application in Fiji and South Pacific nations. It is hoped this proposed model can find applicability in the setting of national primary care research agendas.

## Method

### Pre-workshop

A steering committee comprising a member of WONCA APR council and the President of FCGP garnered sponsorship from Ministry of Health, WONCA APR and pharmaceutical agencies to organize the primary care research workshop in Fiji. They invited a team of three external facilitators from the Asia-Pacific region to organize and operationalize the workshop.

The steering committee proceeded to enlist and engage local physicians (largely from primary care clinics and several from hospitals) from both the public and private sectors, academics from universities and research support officials (including those from research ethics office) from Ministry of Health to participate in the workshop. They also enlisted local public health researchers to co-facilitate the workshop.

Enlisted participants were invited to describe clinical issues encountered in their respective practices or their areas of research interest using a standardized template, highlighting the clinical relevance and significance. They submitted to the Fiji College of General Practitioners (FCGP) for collation and then emailed to the external facilitators.

External facilitators reviewed these submissions to have an overview of the local clinical issues. They used them as resource materials in the workshop to prepare and enable the participants to convert these clinical issues into research questions.

To gain better insight into the healthcare infrastructure, resources and material for research, the external facilitators visited local healthcare institutions, including private solo and group primary care clinics, a public primary care health center and a district hospital. They rode on the opportunity to interact with local healthcare professionals to appreciate and understand the magnitude and clinical significance of endemic healthcare issues, which helped prioritize the key areas of research.

The 3 external and 7 local facilitators (academics, practitioners, local leaders) met for a briefing to identify local research expertise and resource personals to support the novice primary care researchers after the workshop.

### During workshop

Participants were introduced to the scope of primary care research at the workshop with reference to the Larry Green’s primary care research model [6]. The external facilitators converted the participants’ pre-workshop clinical issues into broad potential research questions and framed them into the domains in the Larry Green “Generalist Wheel of knowledge and understanding” [6]. The intent was to enable the participants to appreciate the wider scope of primary care research beyond the domain of clinical research in diseases and treatment to the domains of the patients, their families and the community; the healthcare professionals; health service and policy; as well as topics relating to the interface between domains such as doctor-patient relationship.

The external facilitators incorporated the research topics from the pre-workshop submissions into the workshop modules and presented them to the participants to contextualize the areas of research interests to local setting.

Participants were divided into three teams, each supported by one external and two local facilitators. During scheduled group discussions, each team of facilitators guided the participants through the research process by deliberating on the proposed clinical issues, refining them into answerable research questions, choosing appropriate research design, and developing the research idea into a feasible research proposal.

The external facilitators provided participants with opportunities to highlight other or new research ideas and proposals during informal sessions or refreshment intervals. The participants grouped themselves to form teams for each proposal.

The team representatives presented their respective proposals at day 2 of workshop and invited comments and critique from peers amongst the audience, with feedback from the external facilitators on the content of the proposal and input about logistic feasibility from the local facilitators. Each presentation included (1) the relevance and significance of the research question and topic in relation to gaps in the local healthcare context, and (2) the appropriateness of the methodology for each respective research question.

The external facilitators collated the initial research proposals, assembled them into a preliminary research agenda, analyzed the content and classified them into h the domains of the Generalist Wheel of Knowledge.

## Results

The range of 17 topics proposed by participants collated by external facilitators before the workshop is presented in Table 1. Table 2 depicts the 6 short-listed proposals developed and presented by teams of participants on Day 2 of the workshop. The external facilitators grouped the research topics into four research domains based on Larry Green’s Generalist Wheel of Understanding, in Tables 1 and 2 [6].

**Table 1:** **Clinical issues proposed by the participants before the workshop**

**Clinical areas**	**Specific clinical issues**	**Research domains** ^**#**^
Maternal and child health	Effectiveness of CTG in the monitoring of maternal labor during childbirth	Disease
	Diagnostic investigations for rheumatic health diseases amongst affected children; issues relating to referral to secondary care	Disease
Communicable diseases	Management of patients with undifferentiated symptoms in early phase of dengue, typhoid and Leptospirosis.	Disease
	Inappropriate use of antibiotics for viral infection such as dengue	Disease
	Accuracy of simple test to diagnose dengue using tourniquet in resource-poor community	Disease
	Management of hemoptysis amongst patients with pneumonia	Disease
Non-communicable diseases	Rising prevalence of hypertension and diabetes mellitus: managing at risk patients with these chronic diseases in rural communities	Disease
	Management of diabetic-related complications in primary care	Disease
	Perception of diabetic patients for their foot care	Patient
	Management of type 2 diabetes mellitus: evidence-based pharmacotherapy	Disease
	Physician management of hypercholesterolemia amongst diabetic patients	Disease
	Management of acute asthma exacerbation: effectiveness of pharmacotherapy at an emergency setting	Disease
	Effectiveness of evidence-based pharmacotherapy to achieve and maintain asthma control	Disease
Trauma and injuries	Epidemiology of road traffic accidents in Cook Islands	Disease
Alternative medicine	Impact of Livomyn on the liver function amongst kava drinkers	Disease
Training of primary care physicians	Patients’ perception of the continuous medical education of their general practitioners	Patient
Medical defense for primary care physician	Mandatory implementation of medical indemnity for general practitioners in Fiji	Physician

^#^Research Domains as classified in the “Generalist Wheel of Knowledge” [6].

**Table 2:** **Research proposals presented by the participants during the workshop**

**Potential lead**	**Team members**	**Research question**	**Proposed research method**	**Research domain** ^**#**^
Academics and researchers from university and MOH	GPs from singleton and group practices	What is the prevalence of depression amongst Fijians aged 18 to 40 years who are managed in primary care?	A questionnaire survey of Fijians who consulted primary care clinics	Disease
Academics and researchers from university and MOH	Researchers from MOH (Western division)	What is the prevalence of Yaqola (*Piper methysticum*) consumption among high school students in the Western Division?	A questionnaire survey of high school students in Fiji	Disease
Fiji College of General Practitioners	GP and primary care physician (PCP) in public health centers in Fiji	Amongst primary care physicians (both in public and private sectors) in Fiji, what are their treatment targets for patients with type 2 diabetes mellitus (T2DM)?	A cross-sectional survey of PCPs in Fiji who managed T2DM patients	Physician
Primary care physician	PCP in public health centers in Vanuatu and academic from Fiji	What is the association between air pollutants from volcanic eruption and the health of residents on Tanna Island in Vanuatu?	A prospective study of patients who present with acute respiratory conditions in one hospital and 9 health centers on Tanna Island in relation to local volcanic pollution indicator	Disease
Primary care physician	PCPs from health centers and medical officers from hospital	What is the correlation between the asthma exacerbation rate and pollution from the burning of sugarcane plantation?	Prospective study of asthma patients in Ba, Cakaudrove and Macuata district on Viti Levu island in Fiji	Disease
Ministry of Health	Medical officers from hospitals and researchers from disease-specific public health agency	What are the factors associated with diagnostic and treatment delays of patients with active tuberculosis?	A retrospective study of tuberculosis patients who are managed in DOTS centres in Fiji	Primary health care and system
Fiji College of General Practitioners				

^#^Research Domains as classified in the “Generalist Wheel of Knowledge” [6].

## Discussion

### Summary of main ideas

This article describes the development of a national primary care research agenda through collaborative efforts by local stakeholders and external facilitators. Tables 1 and 2 show the breath of topics highlighted by the participants, which can be potentially incorporated into the research agenda. However time constraints at the workshop limited the number of research proposal presentations by the teams, which explained the reduction in the number of presented proposals.

The external facilitators condensed the research agenda into four main research domains for ease of conceptualizing, understanding and further communication with local health authority for the latter’s support to actualize the research endeavor [6]. The research agenda also gained a broader perspective using this framework to avoid a skewed focus on clinical research and to ensure a more equitable resource distribution to support research in the other domains. However, the lack of presentation of any proposal focusing on the patient domain is noteworthy, despite such proposal being submitted in the pre-workshop (refer to Table 1). The authors postulated that time constraint and dynamics of the team and their selection of presenter at the workshop could be possible reasons.

What is novel is that it presents a plausible model for understanding of the processes involved in the creation of a national primary care research agenda. The processes involved can be categorized into six essential stages, starting with the alphabet E (Table 3).

**Table 3:** **Developing a research agenda using the six “E” steps**

**Stage**	**Process**	**Illustration**	**Stakeholders**
Pre-workshop	Entreat	Local FM leaders form steering group actively seeks official sponsorship from the local Ministry of Health and concerted support from local primary care physicians from the College of General Practitioners and academic staff from FM department in local university. Steering group also entreats external primary care organization such as WONCA to identify regional expertise and sponsor external facilitators to operate the workshop.	College of GP, MOH, Academic institution, WONCA experts
	Enlist	The steering group identifies and puts up a list of primary care physicians from both public and private health sectors with research inclination to be potential participants in a primary care research workshop.	Primary care researcher
	Engage	The steering group members engage academic staff and established researchers from academic institutions to co-facilitate the research workshop and to provide input on local resources and expertise to support research. The external facilitators also engage and partner the local researchers and resource personnel (to identify funding sources and to direct to relevant approval agencies such as ethical committee for research) to define specific roles at the workshop and streamlines the program at a pre-workshop briefing. This is to ensure that the program is contextualized to the local healthcare and academic system.	Academic institution, MOH, WONCA experts
At workshop	Educe	External facilitators educe the participants to identify key local healthcare issues and form research questions and develop respective research proposals. Participants are encouraged to form research teams to further develop their respective proposals.	WONCA experts, primary care researcher
	Empower	External facilitators train the participants to apply appropriate research design to answer their research questions in their proposal. Local facilitators introduce and highlight local resources and expertise to participants who can potentially assist and support the latter when they proceed with their research project. Participants present and share their proposals to each other for clarifications and mutual understanding.	WONCA experts, MOH, primary care researchers
	Encapsulate	External facilitators collate the research proposals, organize them into a research agenda and to seek advice from the local FM steering group regarding feasibility. This is followed by classification into broad research domains and identification of key drivers to lead specific areas of research. Lastly, the external facilitators and the workshop participants agreed on the next steps and ways to operationalize the research proposals.	WONCA experts, primary care researchers

WONCA: World Organization of National Colleges, Academies and Academic Associations of General Practitioners/Family Physicians.

MOH: Ministry of Health (or National Department of Health equivalent in the respective country).

This model uses a logical and sequential framework to illustrate its development, with the local steering committee members played an active role in the first three stages as part of the pre-workshop preparation. The external facilitators anchored the remaining three stages during the workshop execution.

It began with “Entreat” for the steering workgroup members to secure support and sponsorship from governmental agency to fund the workshop. The next stage involved their enlistment of the key primary care stakeholders to participate in the workshop, followed by their engagement of external and local facilitators to execute the workshop.

During the workshop, the external facilitators educed the participants to identify major healthcare issues of clinical relevance and significance in the local community. They empowered the participants with the relevant research skills and guided them to use the appropriate research methodology to bridge the highlighted gaps. During this stage, the local facilitators also availed the participants to the funding resources and expertise to support their research projects. Finally the external facilitators encapsulated the variety of research proposals developed during the workshop into easily assimilated research domains (disease, patient, healthcare professionals, and health system) and developed concrete plans with the participants on ways to operationalize the research proposals.

### Application for future research

The model can serve as a template for other nations who are planning to develop their unique community-centric primary care research agenda. It is configured into six steps, with three as pre-workshop processes and the next three during the workshop. Further evaluation is needed to assess its ease of implementation when opportunity arises for other countries with such intent.

### Limitations of this study

Whether primary care research continues to progress will depend on a multitude of factors such as the dynamics and perseverance of the research teams, their access to adequate consultancy from local experts and support and sponsorship from the official and other funding agencies. Nonetheless, the participants are primed during the workshop to continue their efforts to translate the proposals into actual projects. The steering group and local facilitators will play a catalytic role to facilitate this effectuation of the projects and evaluate the progress of their implementation.

## Conclusion

The proposed “six E” model, consisting of “entreat”, “enlist”, “engage”, “educe”, “empower”, and “encapsulate”, defines the development of the research agenda and provides a staged model that is visible and translatable in building a country’s primary care research agenda.

## Abbreviations

WONCA: World Organization of National Colleges, Academies and Academic Associations of General Practitioners/Family Physicians
APR: Asia Pacific Region
MOH: Ministry of Health
FCGP: Fiji College of General Practitioners
GP: General practitioner
